# Surgical management of osteoporotic pelvic fractures: a new challenge

**DOI:** 10.1007/s00068-012-0224-8

**Published:** 2012-09-23

**Authors:** P. M. Rommens, D. Wagner, A. Hofmann

**Affiliations:** Department of Trauma Surgery, Center of Musculoskeletal Surgery, University Medical Center, Johannes Gutenberg University, Langenbeckstrasse 1, 55131 Mainz, Germany

**Keywords:** Pelvis, Pelvic ring, Osteoporotic fracture, Insufficiency fracture, Classification, Treatment, Osteosynthesis, Transsacral bar, Angular stable plate, Iliolumbar fixation

## Abstract

The number and variety of osteoporotic fractures of the pelvis are rapidly growing around the world. Such fractures are the result of low-impact trauma. The patients have no signs of hemodynamic instability and do not require urgent stabilization. The clinical picture is dominated by immobilizing pain in the pelvic region. Fractures may be located in both the ventral and the dorsal pelvic ring. The current well-established classification of pelvic ring lesions in younger adults does not fully reflect the criteria for osteoporotic and insufficiency fractures of the pelvic ring. Most osteoporotic fractures are minimally displaced and do not require surgical therapy. However, in some patients, an insidious progress of bone damage leads to complex displacement and instability. Therefore, vertical sacral ala fractures, fracture dislocations of the sacroiliac joint, and spinopelvic dissociations are best treated with operative stabilization. Angular stable bridge plating, the insertion of a transsacral positioning bar, and iliolumbar fixation are operative techniques that have been adapted to the low bone mineral density of the pelvic ring and the high forces acting on it.

## Introduction

Pelvic ring fractures typically result from high-energy trauma. A force between 2,000 and 10,000 newtons is required to disrupt an adult pelvic ring [1]. Such high forces are generated in traffic accidents, crush traumas, and falls from great heights. Very often, soft tissues inside the small pelvis and around the pelvic ring are also disrupted [2]. Classification is related to the direction from which the impact on the pelvic ring came and the degree of instability. Treatment consists of an emergency stabilization, which is part of the resuscitation protocol, and a definitive fixation. The type of definitive treatment depends on the localization of the lesions and the amount of instability. Today, because of increasing life expectancy, we are confronted with a growing incidence of both osteoporotic and insufficiency fractures of the pelvic ring [3, 4]. Their mechanism of trauma, symptoms, and treatment differ from those of other types of adult pelvic ring injuries. Osteoporotic and insufficiency fractures result from low-impact trauma, and multiple injuries are rare in these patients. In most cases, conventional radiographs can detect only ventral pelvic fractures; therefore, the severity of the injury is often underestimated. Additional computed tomography (CT) examinations are necessary to evaluate the dorsal pelvis. Fracture patterns in the dorsal pelvis range from crush lesions in the lateral sacrum to spinopelvic dissociations. This manuscript provides an overview of osteoporotic pelvic ring insufficiency fractures and describes their spectrum of instability. New treatment strategies adapted to the specific problems of osteoporotic bone and high strain are presented.

## Pelvic ring injuries in adolescents and adults

The pelvic ring is the strongest and largest osteoligamental complex of the human body. It is divided into the dorsal and ventral hemipelvis. In the dorsal hemipelvis, the sacrum is connected to the iliac bones by the ventral, interosseous, and dorsal ligaments. The body weight is transferred from the lumbosacral spine via the dorsal hemipelvis to the hip joints and the lower extremities. In the ventral hemipelvis, the pubic bone and ischium are connected at the symphysis pubis. The loading forces on the ventral hemipelvis are lower than on the dorsal hemipelvis [1]; accordingly, the bones in the ventral hemipelvis are smaller and more easily broken. To disrupt an adult pelvic ring completely, forces as high as 10,000 newtons are needed [1]. These forces are only generated during high-velocity trauma; hence, these patients are usually polytraumatized. They require urgent resuscitation and early stabilization of their major musculoskeletal instabilities. Pelvic sheets and binders are used during transport from the accident site to the hospital, and pelvic C-clamps and external fixators are required in the shock room or operation theater. These stabilizers have the common goal of provisionally reducing the pelvic ring. Reducing instability enables blood clotting and reduces blood loss in the small pelvis.

The radiological diagnostic work-up consists of three pelvic overviews in which pelvic fractures can be excluded or confirmed. In the anteroposterior view, pubic and ischial rami fractures and symphysis pubis disruptions can be recognized easily. Large dislocations and fracture–dislocations of the dorsal hemipelvis are also assessable. Non-displaced or slightly displaced rami fractures can sometimes be overlooked. In the pelvic inlet view, even small rotatory displacements can be diagnosed. Crush zones in the sacral ala are also visible. The vertical displacement of the disrupted hemipelvis is best observed in the pelvic outlet view, and changes in the sacral body and neuroforamina are also easily observed in this view. A thorough analysis of the dorsal sacroiliac complex is often hindered by abdominal fat or bowel content. Sometimes, fractures are suspected but cannot be confirmed. Therefore, a CT scan of the pelvic ring is compulsory in all cases in which fractures are suspected or confirmed. In fact, total-body CT scans are becoming the standard of care for the emergency diagnosis of all polytraumatized patients [5]. Two- and three-dimensional reconstructions may add to the information from transverse CT slices. Magnetic resonance imaging (MRI) examinations are of little value in the acute diagnostic work-up of pelvic disruptions. MRIs are more useful for evaluating chronic or progressive pain in the pelvic region in the non-trauma setting [6–8].

The classification systems of Burgess [9] and Tile [10] consider the direction of the traumatizing energy, along with the degree of instability. Fracture morphology and localization are important, but they are not the only factors that characterize a pelvic trauma. Ligamentous ruptures, which are associated with pelvic ring lesions, are the most important injuries that have a major influence on stability. In cases of external rotational instability (“open-book injury”), the ligaments and soft tissue structures of the pelvic bottom and the sacroiliac ligaments are partially torn. In vertically unstable fractures, all ligaments of the dorsal pelvis and the pelvic bottom are torn.

During high-energy trauma to the pelvis, other soft tissue structures are often damaged [11]. Blood loss results from bleeding in the large fracture parts of the dorsal iliac bone and sacrum and the venous plexus in the small pelvis. Disruptions of the urethra and the bladder are often seen with rotational and vertically unstable lesions; rectal and vaginal tears are less common [2]. Degloving injuries and Morel-Lavallé lesions are seen in crush traumas [12, 13]. Nerve injuries are diagnosed in severely displaced fractures or dislocations of the dorsal pelvis and in fractures of the central part of the sacrum [14–16]. All of the above-mentioned lesions are a direct sign of the severity of the pelvic injury and have a major influence on the long-term prognosis.

Therapeutic principles depend on the localization and the extent of instability. While isolated fractures of the pubic and ischial rami are treated conservatively, ruptures of the symphysis pubis and fractures or dislocations of the dorsal pelvic ring are treated surgically. Osteosynthetic techniques rely on different but well-known principles: compression, neutralization, and bridging. Symphysis pubis ruptures are reduced and bridged with a plate osteosynthesis, while sacral fractures and dislocations of the sacroiliac joint are treated with compression sacroiliac screw osteosynthesis. Fractures of the iliac bone are reduced and fixed with lag screws along the iliac crest and neutralization plates along the pelvic brim. The above-mentioned therapeutic principles are used in combination, depending on the fracture pattern [17, 18]. In patients with normal bone quality, implant-related problems such as loosening, cutting out, or implant failure are rare.

## Osteoporotic and insufficiency fractures of the pelvis

Because of increasing life expectancy, the number of elderly patients is rapidly growing. Many of the elderly patients are still healthy, physically very active, and have high functional demands. Others have multiple comorbidities, lower functional demands, and may even be bedridden. Both groups suffer pelvic osteoporotic and insufficiency fractures [3, 4, 19, 20], and the treatment goal for both groups should be restoring the level of function they had before the injury.

### Patient history

**Fig. 1:**
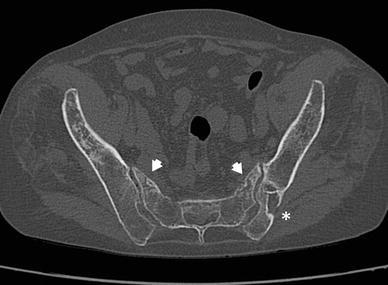
Bilateral fracture of the sacral ala in a 79-year-old patient with previous lumbar spine fusion surgery (*arrows*). The site of bone harvesting is near to the left sacroiliac joint and to the left sacral fracture (*asterisk*)

Bone density and strength are reduced in most of these patients. They have osteoporosis and vitamin D deficiency, have taken long-term courses of cortisone medication, were irradiated on the small pelvis for a malignant disease, underwent cancellous bone harvesting in the dorsal pelvis for lumbar spine fusion operations, or were bedridden for long periods for other reasons [21–25] (Fig. 1). In these patients, pelvic fractures occur after a low-impact trauma, such as a fall from the standing position, from a chair in a sitting position, or out of bed. In some patients, the trauma history is not memorable. This traumatic energy is too low to cause a pelvic ring fracture in a healthy adult. Patients may complain of pain in the groin, dorsal pelvic pain at the sacroiliac joints, or lower back pain [26]. Sometimes, patients experience a pseudoradicular pain radiation beyond the knee. Many patients are unable to walk; others may only be able to walk with the help of walking devices.

### Clinical and radiological examination

**Fig. 2:**
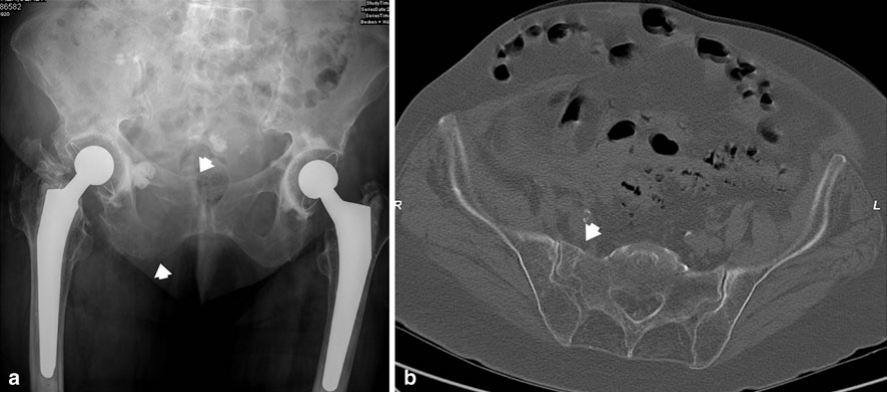
**a** Pelvic overview of 91-year-old female with severe osteoporosis. Pubic and ischial rami fractures with slight displacement are visible (*arrows*).** b** Transverse CT-cuts through the sacrum showing a vertical fracture in the sacral ala (*arrow*). The patient was treated conservatively

**Fig. 3:**
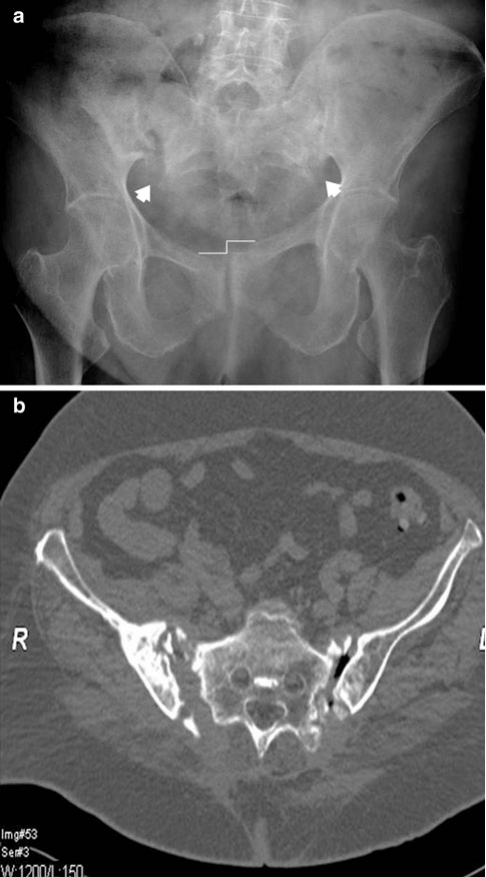
**a** Bilateral fractures of sacral ala with intrusion of the lumbar spine and sacrum into the small pelvis. There also is instability of the symphysis pubis.** b** Transverse CT-cuts through the sacrum and dorsal ilium, showing bilateral insufficiency of the sacroiliac joints with widening of the right joint and nitrogen bubble in the left joint

**Fig. 4:**
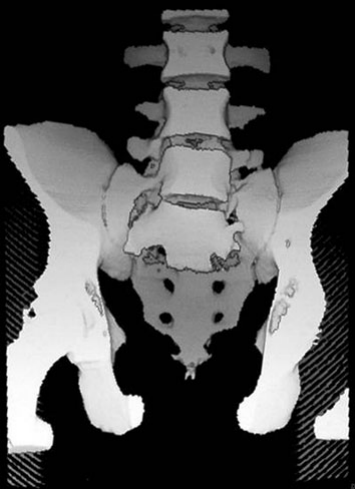
3D-Reconstruction of a suicide jumper’s fracture in an 18-year-old male. There is complete spinopelvic dissociation, separating the body of S1 from the rest of the sacrum

**Fig. 5:**
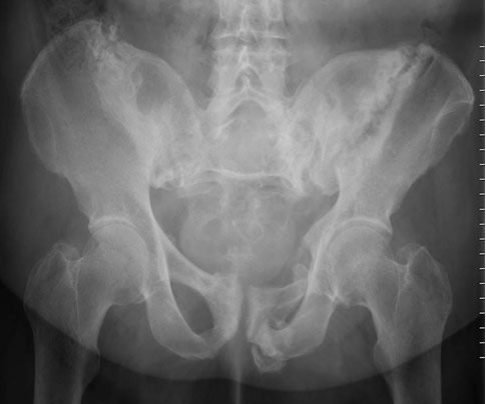
Bilateral ilium fractures, starting at the inner curves of the iliac wings, in a 67-year-old female patient with severe osteoporosis. A slight intrusion of the lumbar spine and the sacrum in the small pelvic ring is visible. There also are pubic and ischial rami fractures on the left and instability of the symphysis pubis

Many patients suffer from spontaneous pain of the symphysis pubis or pain on local pressure. Others have additional pain in the dorsal hemipelvis, the sacral body, or the sacroiliac joints. When manual pressure is simultaneously placed on both iliac crests, patients report severe pain in both the dorsal and ventral hemipelvis. Radiological examinations are conducted in order to confirm suspected fractures. The three conventional views (pelvic AP, inlet, and outlet views) are the first step of the diagnostic work-up to detect pubic and ischial rami fractures, symphysis pubis disruptions, and rotational and vertical displacements. The diagnosis of nondisplaced or incomplete lesions is much more difficult, especially in the dorsal pelvic ring [27, 28]. Special attention should be given to the sacral ala, as most lesions are located there [29]. Because dorsal pelvis visualization is limited with conventional views, we recommend performing CT imaging for all patients for whom pelvic injuries are suspected. Sometimes, coronal reconstructions are more useful than transverse cuts for analyzing the dorsal hemipelvis. Fractures of the sacrum are typically situated at the sacral ala, between the neuroforamina and the sacroiliac joints [29, 30] (Fig. 2a, b). They can be fissures, nondisplaced, or displaced fractures. In chronic cases, a destruction of the cancellous bone with widening of the fracture gap is visible. The fracture can also extend into the sacroiliac joint. Nitrogen bubbles in the sacroiliac joint represent an instability of this joint (Fig. 3a, b). Bilateral vertical sacral lesions can be connected by a horizontal fracture line. In these cases, the vertebral bodies S1 or S1 and S2 are completely separated from the rest of the pelvic ring. The fracture morphology is similar to that of spinopelvic dissociation or suicide jumper’s fracture in the younger adult [31, 32] (Fig. 4). In other cases, fractures start in the sacroiliac joint and extend into the dorsal ilium, similar to the path of crescent fracture of the lateral compression injury in the younger adult. Fractures may also start at the inner curve of the iliac wing (Fig. 5). In the ventral hemipelvis, lesions can have different morphologies and localizations. Nondisplaced or slightly displaced fractures of the pubic and ischial rami are the most common types. In chronic cases, erosion of the fracture margins with callus formation and widening of the fracture gap is visible. Similarly, chronic instability of the symphysis pubis is characterized by marginal erosion and marginal callus formation. In a few cases, the origin of pelvic pain remains unclear after conventional and CT examinations. In these cases, MRI of the pelvis is recommended so as to exclude other reasons for low back and pelvic pain. A bone bruise in the sacral ala can sometimes be seen. We believe that such bone bruises correspond to the onset of a disruption of the cancellous structure of the lateral sacrum, and the lesion is the first stage before an insufficiency fracture occurs [6–8].

### Classification

Combinations of different types of lesions of the ventral and the dorsal hemipelvis result in a spectrum of instability. Nevertheless, they can only be partially classified with the Burgess [9] or Tile [10] classification systems, as there are distinct differences between the characteristics of lesions in the elderly and in younger adults. Unlike younger adults with pelvic fractures, elderly patients with insufficiency fractures do not have lesions of the pelvic ligaments because the bones are not as strong as the ligaments. The fracture mechanism of insufficiency fractures is comparable to the implosion of a building: the most important forces come from the inside but are triggered by smaller forces coming from the outside. In contrast, the trauma mechanism of open-book and vertically unstable pelvic lesions in younger adults is comparable to an explosion and results from major forces coming from the outside. Lateral compression lesions in younger adults and the elderly are similar in morphology: both patient groups present pubic rami and ischial fractures in the ventral hemipelvis in combination with crush zones in the sacral ala and crescent fractures. At their most extensive, pelvic osteoporotic or insufficiency fractures are similar to spinopelvic dissociations [29, 31, 32]. The vertebral bodies of S1 or S1 and S2 remain in continuity with the lumbar spine but are disconnected from the pelvic ring. In chronic cases, there is slight intrusion of the lumbosacral spine into the small pelvis. This fracture pattern is not identified in the classification systems of adult pelvic trauma. Although vertically unstable, these lesions are not type C lesions because the ligaments of the pelvic floor and the dorsal iliosacral complex are intact. We assume that the natural history of many of these lesions is one of insidious but continuous progress. Triggered by repetitive smaller traumas, increasingly more bony structures are damaged, leading to more complex fracture patterns and greater instabilities. The process begins with a slightly displaced pubic rami fracture and a nondisplaced fracture in the lateral sacrum and then develops into a spinopelvic dissociation when inadequately treated.

## Treatment strategies

Because osteoporotic pelvic fractures are the result of low-impact trauma, the patient’s clinical picture upon arrival at the emergency department or outpatient clinic differs from that of a younger adult patient with pelvic ring disruption. Immobilizing pain is the most significant symptom, and hemodynamic instability is not present. For this reason, emergency stabilization with pelvic sheets, binders, C-clamps, or external fixators is not necessary [33, 34].

Most osteoporotic and insufficiency fractures of the pelvis can be treated conservatively. They are minor lesions with little instability. Treatment consists of bed rest and pain medication, followed by mobilization out of bed and partial weight-bearing of the injured side. The degree of osteoporosis and bone metabolism should be investigated, and adapted drug therapy should begin [35, 36]. As soon as the pain intensity diminishes, weight-bearing is increased until full weight-bearing is achieved. When intense pain persists or increases, repeated pelvic overviews or additional CT examinations are recommended in order to exclude fractures or displacements that may not have been visible or present at admission. Pain can persist for as long as six to eight weeks after the trauma.

Pubic and ischial rami fractures with greater displacement are generally associated with dorsal instability [27, 28]. If an incomplete or complete nondisplaced sacral fracture is detected, surgical treatment should be considered. Stabilization significantly diminishes the pain intensity and allows earlier patient mobility. Percutaneous screw fixation is possible in most cases [37, 38].

In cases of displaced dorsal fractures or fracture–dislocations, surgical treatment is inevitable. With bed rest only, bony healing will not occur. The patients will remain bedridden for a lengthy period and will suffer from such complications as pressure sores, pneumonia, urinary infection, and muscle weakness, a syndrome previously known as “fracture disease”. The appropriate operative technique will depend on the morphology and localization of the instability [39].

### Osteosynthesis of the ventral hemipelvis

**Fig. 6:**
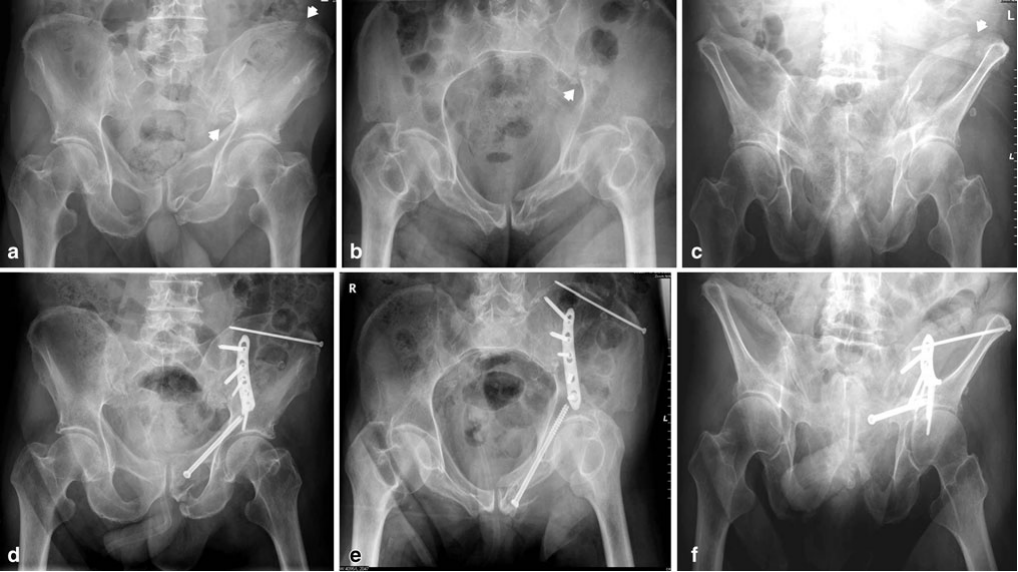
** a** Left ilium fracture and left pubic rami fractures in a 78 year old male with Alzheimer’s disease and after repetitive falls at home. The disruption and gap at the iliac crest is clearly recognizable (*arrows*).** b** Pelvic inlet view, showing the slight internal rotation of the left hemipelvis. The exact place of the cortical disruption at the pelvic brim is also visible.** c** Pelvic outlet view, also showing the fracture and displacement of the iliac wing.** d** Postoperative pelvic ap overview. The gap at the iliac crest has been closed and fixed with a long lag screw. An angle stable plate has been placed along the sacroiliac joint and over the pelvic brim, bridging the fracture area. In the anterior pelvis, a retrograde pubic screw has been placed.** e** Postoperative pelvic inlet view.** f** Postoperative pelvic outlet view. There was an uneventful healing. Patient was again able to ambulate independently

**Fig. 7:**
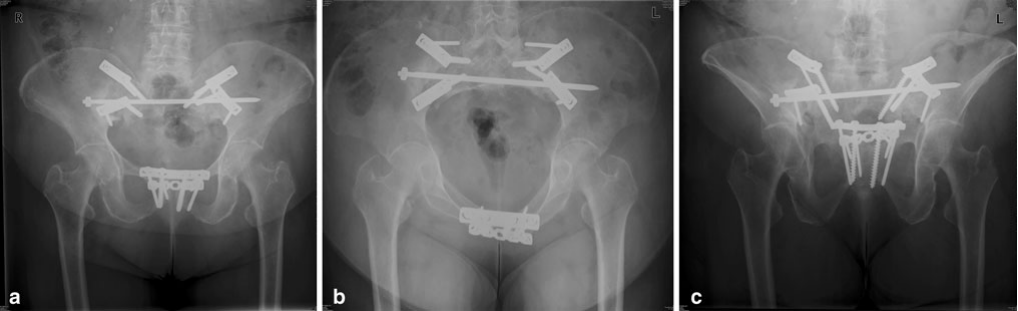
**a** Postoperative ap pelvic overview of the same patient as in Fig. 3a–b. A bilateral fusion with debridement of the sacroiliac joints, cancellous bone grafting, double anterior plate osteosynthesis over the sacroiliac joint and transsacral bar osteosynthesis were performed to restore stability in the dorsal pelvis. At the symphysis pubis, a double bridging plate osteosynthesis was also performed.** b** Postoperative inlet view, nicely showing the intraosseous trajectory of the transsacral bar. **c** Postoperative outlet view. Two years after surgery, the patient is able to walk smaller distances without walking devices and help of other people

The type of osteosynthesis depends on the precise localization of the lesion. Instabilities of the symphysis pubis are fixed with a bridging plate osteosynthesis [40]. In this osteoporotic bone, a six-hole angle-stable plate is recommended. In patients with high body mass index, a double-plate osteosynthesis should be considered (Fig. 7a–c). In this case, one plate is placed on the superior edge of the symphysis, and the other plate is placed anteriorly. In cases of pubic bone fractures very close to the symphysis, the same procedure is performed. In more lateral fractures, a longer plate is slipped below the iliac vessels. The most lateral screws are drilled just medial to the acetabular cavity from the anterior down to the posterior column. The long trajectory of these screws significantly adds to the stability of the construct. Furthermore, screws can be placed posteriorly to the acetabulum through the modified Stoppa approach. In cases of fractures in the isthmus of the pubic ramus, a retrograde “intramedullary” pubic screw is a good choice (Fig. 6a–f). The fracture parts are aligned and fixed with a large-diameter screw, which is inserted at the pubic tubercle and passes medially and cranially from the acetabulum. The screw can have a length of up to 100 mm. If the fracture is situated very close to the acetabulum or passes through the anterior lip of the acetabulum, the same procedure can be performed [41]. Bilateral lesions are treated with bilateral retrograde screws. External fixation with supra-acetabular pins is not a good option in elderly patients [28, 37]. The fixator pins must remain in place for several weeks, until the fracture fragments are bridged by a callus. The lack of patient comfort together with a high risk of pin loosening, pin-track infection, and soft tissue breakdown are strong arguments against this type of ventral fixation. Alternatively, a temporary internal fixator can be used with supra-acetabular pedicle screws and a subcutaneous connecting bar. However, in addition to implant removal, a high incidence of ectopic ossifications and lesions to the lateral femoral cutaneous nerve is observed [42].

### Osteosynthesis of the dorsal pelvis

Different therapeutic principles can be used to reduce and/or fix dorsal instabilities in insufficiency fractures of the elderly. These principles will be discussed, along with their respective advantages and limitations.

#### Iliosacral screw osteosynthesis

Iliosacral screw osteosynthesis is a valid option for the fixation of sacrum fractures and sacroiliac joint dislocations in the elderly, as it is for similar lesions in younger adults. Screws can be inserted percutaneously, with the patient in the supine or prone position [38, 43]. Ideal indications for a percutaneous procedure are nondisplaced sacral ala fractures. The prone position is indispensable when an open reduction of the dorsal instability is required. To avoid any rotational instability, two screws are inserted in the vertebral body of S1 [44]. Because the density of the cancellous bone is higher in the central part of the sacrum than in its ala, the tip of the screws should reach the midline [45]. Moderate compression can be placed on the fracture by tightening the screw. Placing a washer below the screw head prevents the screw head from perforating the lateral cortex of the dorsal ilium. Nevertheless, the low purchase of the screws in the osteoporotic bone presents a high risk of loosening. To avoid this, cement augmentation of iliosacral screws has been recommended [46].

#### Sacroplasty

This technique is increasingly used by radiologists to treat sacral ala fractures. Smaller series published in radiological and spine journals present cases of nondisplaced fractures, for which this technique has been used [47–61]. The technique relies on the same principles as vertebroplasty. A small amount of bone cement is inserted in the fracture area. By the force of application, the fluid cement is distributed throughout the cancellous bone around the fracture site. The fracture site is stabilized as soon as the cement hardens. In documented case series, pain relief is significant and early mobilization of patients is possible; however, the published experience is too limited to recommend this method as a standard of care. In our opinion, this method should only be used for nondisplaced sacral ala fractures. In displaced fractures or chronic cases, the cement acts as a void filler but not as a stabilizer and, as we suppose, inhibits bony healing at the fracture site. The cement will hinder later surgery, especially iliosacral screw placement if it becomes necessary. Biomechanical studies are needed in order to validate the role of sacroplasty. In vertebroplasty, the bone cement must be able to withstand axial compression forces to prevent further collapse of the vertebral body. The fracture area or zone of compression is situated in the horizontal plane. In sacroplasty, vertical sacral fractures are treated with cement augmentation. How shearing forces work on this bone–cement–bone construct has not yet been clearly shown [30, 53]. In a recent biomechanical study, three methods of fixation of osteoporotic fractures of the sacral ala (sacroplasty, short iliosacral screw, long iliosacral screw) have been compared. Although no significant differences have been found between the groups, there was a tendency of enhanced motion in the sacroplasty group [62]. Furthermore, a combination of an iliosacral screw and the sacroplasty is described, with good results. However, a leakage of cement into the sacral foramina and throughout the fractures should be prevented by the use of a new cement generation [63].

#### Internal fixator

The advantages of internal fixators have been outlined in numerous biomechanical and clinical observational studies. Of special interest is the great holding power of the internal fixator construct in osteoporotic bone. This method is widely used for fractures related to metaphyseal lesions of the humerus, femur, and tibia. Internal fixators are not yet frequently used for pelvic fractures. There are no anatomical locking plates for specific use in the pelvic ring; instead, the plates used for long bones are bent and torn to adapt to the pelvic anatomy. Such plates are used as a bridging implant when inserted between the two dorsal iliac crests at the level of the inferior posterior iliac spines [64, 65]. This osteosynthesis is performed in adult patients with comminuted sacral fractures that are part of vertically unstable pelvic ring lesions. In this configuration, high stability is obtained in the dorsal pelvis, enabling early mobilization and contributing to quick healing, providing that the anterior pelvic ring is also reduced and fixed. The plate acts as a bridging and locking implant, but no compression is generated in the fracture areas. The construct does not guarantee the neutralization of shear forces in bilateral sacral ala fractures, fracture–dislocations of the sacroiliac joint, or spinopelvic dissociations. We, therefore, recommend using a locked dorsal bridging plate as an additional stabilizer after the placement of iliosacral screws, which close the fracture gap and create compression within it.

Internal fixators can also be used inside the pelvic ring. They are placed along the sacroiliac joint and more distally over the inner curve of the ilium. The locking plates must be bent and twisted accordingly. Indications are fracture–dislocations originating in the lower part of the sacroiliac joint and extending proximally and laterally into the dorsal ilium (crescent fracture) or ilium fractures that originate in the sciatic notch and extend proximally and laterally through the iliac wing towards the iliac crest. The fracture is exposed and reduced on the inside of the iliac wing. A long position screw is placed along the reduced iliac crest. The most proximal screws of the internal fixator are inserted in the posteromedial direction, parallel to the sacroiliac joint; the distal screws are directed strictly posteriorly or posterolaterally along the inner curve of the ilium. The internal fixator acts as a strong bridging construct with a high purchase, preventing pull-out or secondary fracture fragment displacement (Fig. 7a–f).

In the ventral pelvis, small-fragment angle-stable symphysis plates are often used. In patients with a high body mass index, a large-fragment angle-stable plate is used or double-plating is performed.

#### Transsacral positioning bar

A long-threaded 6-mm bar is positioned from one dorsal ilium through the vertebral body of S1 towards the opposite dorsal ilium. Washers and nuts are placed on both ends of the bar. Tightening the nuts provides compression in the vertical fracture plane in the lateral sacrum [66–70]. Unlike in screw osteosynthesis, the level of compression is independent of the bone quality; rather, it is related to the amount of force the washers place on the lateral cortex of the dorsal ilium. Because the implant is locked on both sides outside the bone, pulling out is not possible. The patient must be placed in the prone position. The ideal entry point on the lateral cortex of the dorsal ilium is identified intraoperatively via the pure lateral view of the sacrum. The canal is drilled with a long 2.8-mm drill bit, which is used for iliosacral screw placement. The drill bit is pushed forward until it perforates the opposite sacroiliac joint and the lateral cortex of the opposite dorsal ilium. The drill bit is left in place and overdrilled with a cannulated 4.5-mm drill bit. After the drill bits are removed, a solid 6-mm threaded positioning bar of adapted length is drilled into place. During drilling, the correct trajectory is controlled using repetitive fluoroscopy in the inlet and outlet pelvic views. On both sides, large washers are placed over the bar. Two nuts are then consecutively placed over the bar; while the medial nut is used for tightening, the second nut locks the position of the first nut [71]. Good indications for transsacral bar osteosynthesis are bilateral vertical sacral ala fractures in combination with anterior pelvic instability or spinopelvic dissociation (Fig. 6a–c). Clinical experience is limited to small series [67–71]. Most patients can be mobilized soon after stabilization and have good functional outcomes. Better-adapted instruments and implants are needed in order to produce a fixation that is less invasive but as strong as what is currently available. In addition, a dysmorphic sacrum should be excluded preoperatively in the CT scan before the decision is made for transsacral bars or screws [72].

#### Iliolumbar fixation

In spinopelvic dissociation, the vertebral bodies of S1 or S1 and S2 are disrupted from the rest of the sacrum and pelvic ring. These first sacral vertebral bodies remain connected with the lumbar spine. Because the strong dorsal iliosacral ligaments remain intact, major vertical instability, as seen in suicide jumper’s fractures in younger adults, is not present here. Nevertheless, the patients have immobilizing pain in the dorsal pelvis and are bedridden unless they are stabilized. The iliosacral spine can protrude into the small pelvis. To prevent the progress of instability, the iliosacral spine must be fixed to the intact dorsal pelvis [73–75]. On both sides, pedicle screws are placed in the pedicles of the third and fourth or fourth and fifth vertebral bodies and in the dorsal iliac crest at the level of the superior posterior iliac spine [76]. The screws are connected with bent bars, and the bars are connected with a small transverse bar. After iliolumbar fixation, a transsacral positioning bar can also be inserted to create interfragmentary compression in the vertical fracture gaps [71].

#### Ventral fusion of the sacroiliac joint with plate osteosynthesis

In some patients, instability is not located in the lateral mass of the sacrum but, instead, in the sacroiliac joint. Because of low bone quality, iliosacral screw fixation may not create enough stability to ensure uneventful healing. In such cases, arthrodesis may be the better alternative. The joint is exposed and opened anteriorly through an incision along the iliac crest. The joint is debrided and filled with autologous cancellous bone grafts taken from the ipsilateral iliac crest. The joint is fixed with two large-fragment dynamic compression plates placed over the sacroiliac joint at an angle of 60° to each other. One cancellous bone screw is inserted into the sacral ala parallel to the sacroiliac joint, and one or two cortex screws are placed in the dorsal ilium near the inner curve of the ilium (Fig. 6a–c).

#### Combination of techniques

Depending on the degree of instability, a combination of several osteosynthesis techniques may be needed to achieve adequate stability for early postoperative mobilization [77, 78]. In the dorsal pelvis, a dorsal internal fixator can be combined with iliosacral screw osteosynthesis, and the transsacral positioning bar can be combined with iliosacral screw fixation. Iliolumbar fixation can be combined with a transsacral positioning bar or with iliosacral screw fixation. Ventral plate osteosynthesis of the sacroiliac joint can be combined with iliosacral screw fixation or a transsacral positioning bar (Fig. 6a–c). In the ventral pelvis, plate osteosynthesis can be combined with retrograde pubic ramus screws if a combined instability is present. The ultimate goal is to establish an adequate rigid fixation for every type of pelvic ring instability.

## Conclusion

Because of higher life expectancy, surgeons are confronted with a rapidly growing number of pelvic osteoporotic and insufficiency fractures. In contrast to pelvic ring fractures in younger adults, pelvic osteoporotic fractures in the elderly result from low-impact trauma. Multiple fracture forms are seen, reflecting a spectrum of instability. Pubic rami fractures are the most common lesions, and they are generally combined with a crush zone in the lateral mass of the sacrum. They can be treated conservatively. Typical lesions of the dorsal pelvis are vertical fractures in the lateral mass of the sacrum. When these patients experience severe or increasing pain during mobilization, a fixation should be considered. Other patients experience an insidious progress of bone damage, which leads to greater instability. Complex forms of instability, such as bilateral sacral fractures, crescent fractures, or spinopelvic dissociations, must be treated surgically. New forms of osteosynthesis consider the specific problems of low bone density and high axial load. Little is currently known about the natural history and results of the surgical management of pelvic osteoporotic and insufficiency fractures [79]. More clinical and biomechanical studies are needed in order to better understand their specific characteristics and to develop adapted operation techniques and implants.
